# Patient safety culture among European cancer nurses—An exploratory, cross‐sectional survey comparing data from Estonia, Germany, Netherlands, and United Kingdom

**DOI:** 10.1111/jan.14177

**Published:** 2019-09-04

**Authors:** Lena Sharp, Kristi Rannus, Anna Olofsson, Daniel Kelly, Wendy H. Oldenmenger, the EONS RECaN group

**Affiliations:** ^1^ Regional Cancer Centre Stockholm‐Gotland Stockholm Sweden; ^2^ Division of Innovative Care Research Department of Learning Informatics, Management and Ethics Karolinska Institutet Stockholm Sweden; ^3^ North Estonia Medical Centre Tallinn Estonia; ^4^ Tallinn Health Care College Tallinn Estonia; ^5^ School of Healthcare Sciences Cardiff University Wales UK; ^6^ Department of Medical Oncology Erasmus MC Cancer Institute Rotterdam The Netherlands

**Keywords:** cancer nurses, hospital survey on patient safety, nursing, patient safety culture

## Abstract

**Aim:**

To explore the differences in perceived patient safety culture in cancer nurses working in Estonia, Germany, the Netherlands, and the United Kingdom.

**Design:**

An exploratory cross‐sectional survey.

**Methods:**

In 2018, 393 cancer nurses completed the 12 dimensions of the Hospital Survey on Patient Safety Culture.

**Results:**

The mean score for the overall patient safety grade was 61.3. The highest rated dimension was “teamwork within units” while “staffing” was the lowest in all four countries. Nurses in the Netherlands and in the United Kingdom, scored higher on “communication openness”, the “frequency of events reported”, and “non‐punitive response to errors”, than nurses from Estonia or Germany. We found statistically significant differences between the countries for the association between five of the 12 dimensions with the overall patient safety grade: overall perception of patient safety, communication openness, staffing, handoffs and transitions and non‐punitive response to errors.

**Conclusion:**

Patient safety culture, as reported by cancer nurses, varies between European countries and contextual factors, such as recognition of the nursing role and education have an impact on it. Cancer nurses’ role in promoting patient safety is a key concern and requires better recognition on a European and global level.

**Impact:**

Cancer Nursing Societies in any country can use these data as an indication on how to improve patient care in their country. Recognition of cancer nursing as a distinct specialty in nursing will help to improve patient safety.

## Abstract (zh)

**目的:**

探讨在爱沙尼亚、德国、荷兰和英国工作的癌症护士对病人安全文化的认知差异。

**设计:**

探索性横向调查。

**方法:**

2018年,393名癌症护士完成了医院患者安全文化调查的12个维度。

**结果:**

总体患者安全等级的平均分数为61.3。评分最高的维度是“单位内的团队合作”,而“人员配备”在所有四个国家中都是最低的。荷兰和英国的护士在“沟通开放性”、“事件报告频率”和“对错误的非惩罚性反应”方面得分高于爱沙尼亚和德国的护士。我们发现,在12个维度中,有5个维度与患者总体安全等级之间存在统计学上的显著差异:患者总体安全感知、沟通开放性、人员配备、交接和过渡以及对错误的非惩罚性反应。

**结论:**

癌症护士所报告的患者安全文化在欧洲国家之间存在差异,而护理角色的认知和教育等环境因素也会对其产生影响。癌症护士在促进患者安全方面的作用是一个关键问题,需要在欧洲和全球层面上得到更好的认识。

**影响:**

任何国家的癌症护理协会都可以利用这些数据作为改善本国患者护理的指示。认识到癌症护理是护理的一个独特的专业将有助于提高患者安全性。

## 1 INTRODUCTION

Patient safety is defined as the prevention of errors and adverse effects to patients associated with health care (World Health Organisation, 2018). The World Health Organization (WHO) points out that health care has become more effective during recent decades, but also more complex and that these complexities may challenge efforts to improve patient safety as risks may increase.

Recent positive developments in cancer care with improved treatment outcomes also carry with them new patient safety risks. Potent drugs with small therapeutic margins, complex treatment regimens with severe symptom burdens and issues around adherence to treatment are examples of some of these risks (Weingart, Zhang, Sweeney, & Hassett, 2018). Patients with cancer are vulnerable to errors and mistakes can have catastrophic consequences (Weingart et al., 2018).

Research shows that errors occur during all phases of the administration process of cytotoxic drugs (Fyhr & Akselsson, 2012; Keers, Williams, Cooke, & Ashcroft, 2013; Kullberg, Larsen, & Sharp, 2013; Weingart et al., 2018). Cancer nurses have a central role in this process and they are often the last point of contact during this complex process prior to the drugs reaching the patient (Schwappach & Gehring, 2014). Nurses’ actions and risk assessment skills are of great importance. Safe procedures and the correct use of devices are crucial steps in safety promotion (Kullberg et al., 2013; Mattsson et al., 2015), as is the courage to speak up and question when adverse events do occur in practice, including risks and near misses (Schwappach & Gehring, 2014). This requires a safety culture where adverse events can be reported without staff being blamed and if mistakes do occur, lessons are learned. Patient safety culture has been defined as the overall behaviour of individuals and organizations, based on a common set of beliefs and values that are aimed at reducing the opportunities for patient harm (Singer & Vogus, 2013). The terms “patient safety culture” and “patient safety climate” are sometimes used interchangeably. Safety climate has been described as a snapshot of the underlying safety culture (Danielsson, Nilsen, Rutberg, & Arestedt, 2017). The patient safety culture is an important measure in assessing the quality of health care and generally is measured by surveys (Danielsson et al., 2017; Mascherek & Schwappach, 2017). Research has shown that a high patient safety culture is associated with fewer readmission events (Hansen, Williams, & Singer, 2011), fewer medication errors and a reduction in urinary tract infections (Hofmann & Mark, 2006). Better perceptions of overall patient safety and a higher patient safety grade are also associated with nurses with higher levels of motivation (Toode, Routasalo, Helminen, & Suominen, 2015) and more satisfied patients and nurses (Hofmann & Mark, 2006).

### 1.1 Background

Nurses are considered the most trusted profession in many countries (Brenan, 2018; Stephenson, 2018) and have a central role for people affected by cancer because they represent the largest group of healthcare professionals in the cancer workforce. A recent systematic review (Charalambous et al., 2018) concluded that the contribution of cancer nursing to interventions to benefit patients and to cancer research more generally is significant but is not always recognized. The recognition of cancer nursing as a specialty across Europe is highly variable at present. The RECaN project (Recognising European Cancer Nursing) (Campbell et al., 2017; Charalambous et al., 2018; Kelly & Charalambous, 2017) has been initiated and conducted by the European Oncology Nursing Society (EONS) and supported by the European Cancer Organisation (ECCO). The overall goal is to increase recognition of the value and contribution of cancer nursing across Europe. This exploratory study is a part of the second phase of the RECaN project, where the patient safety culture in cancer nursing is compared across four European countries.

## 2 THE STUDY

### 2.1 Aim

The aim of this study was to explore the differences in the perceived patient safety culture in cancer nurses working in four European countries using the Hospital Survey on Patient Safety Culture (HSPSC). More specifically, the objectives were to compare perceptions and aspects of patient safety cultures that were important as described by cancer nurses in Estonia, Germany, the Netherlands, and the United Kingdom. Besides this goal, we also sought to identify those factors most significantly associated with the highest rates of overall patient safety assessments.

### 2.2 Design and ethical considerations

We conducted an exploratory cross‐sectional study to investigate workplace patient safety culture among cancer nurses in four European countries, i.e., Estonia, Germany, Netherlands, and the United Kingdom. The survey was conducted in all four countries during 2017. Research Ethics Committee approval for this study was obtained from the Ethical Review Committee at Cardiff University in the United Kingdom, with country specific approval from the Ethical Review Board of the Erasmus University Medical Center in the Netherlands and the Research Ethics Committee of the University of Tartu in Estonia. In Germany, additional approval was not necessary.

### 2.3 Participants

Eligible participants were cancer nurses from the four countries involved. The data collection was conducted anonymously on a volunteer basis during annual conferences of the National Cancer Nursing Societies in each country, between May ‐ November 2017. During the conferences, all cancer nurses had the opportunity to complete the questionnaire following an invitation from one of the researchers. This was voluntary and the nurses themselves decided whether they wished to participate. A specific database was developed for the purpose of this study and data from all four countries were stored using anonymized codes.

### 2.4 Data collection

The HSPSC assesses staff perceptions of the patient safety culture, including different aspects of safety, medical errors, and incident reporting. The survey is available in all four languages of the participating countries (Hammer et al., 2011; Smits et al., 2012; Smits, Wagner, Spreeuwenberg, van der Wal, & Groenewegen, 2009; Toode et al., 2015; Waterson, Griffiths, Stride, Murphy, & Hignett, 2010). The Agency for Healthcare Research and Quality gave permission to use the HSPSC in all four languages.

The HSPSC consists of some background variables, e.g. professional experience (years), work time (hours per week), primary working area and whether the nurses participating have direct patient contact. However, we could not collect these background data from Germany, since the German version of HSPSC did not include these variables.

The HSPSC includes 42 items covering 12 dimensions of the patient safety culture, with three or four items per dimension (File S1). All items are based on a five‐point Likert‐type scale, from “strongly disagree” to “strongly agree”, or from “never” to “always”. When necessary, prior to statistical analysis, negatively worded items were reverse coded so that a higher score always represented a positive response. The HSPSC also includes two single‐items that provides an “overall patient safety grade”, with a five‐point Likert‐type scale response (from “Failing” to “Excellent”) and a “number of events reported” (Sorra et al., 2018). The overall patient safety grade item was used as an outcome variable in this study (Sorra & Dyer, 2010).

### 2.5 Validity and reliability

The HSPSC has been published and showed acceptable psychometric properties, as factor analyses confirmed the validity of the HSPSC subscales and the questionnaire showed acceptable levels of reliability across the involved countries. Different studies showed a Cronbach’s alpha >.70, which is acceptable, except for staffing (around 0.60 in most studies). All items of the HSPSC correlate significantly with the safety score (Blegen, Gearhart, O”Brien, Sehgal, & Alldredge, 2009; Lee, Phan, Dorman, Weaver, & Pronovost, 2016; Smits, Christiaans‐Dingelhoff, Wagner, Wal, & Groenewegen, 2008).

### 2.6 Data analysis

Prior to the statistical analysis, the scores of negatively worded items were reversed to ensure that higher scores always reflected a more positive assessment of patient safety culture. The dimension scores were then analysed using two different methods. (a) Percentages of positive responses, defined as values “agree” to “strongly agree” and “most of the time” to “always” (Sorra et al., 2018). The single item “overall patient safety grade” was dichotomized into high (“excellent” and “very good”) and low (“acceptable”, “fair”, and “failing”). The single item “number of events reported” was dichotomized into no events reported and one or more events reported. For each item, we calculated the percentages of respondents who answered positively. Then unweighted averages of those percentages were computed for each dimension, resulting in dimension scores ranging from 0–100; (b) The Likert‐type scale was linearly transformed to a 100‐point scale (Scaled score = [(Raw score−min response score)/range of possible response category scores]*100), with the lowest possible value corresponding to 0 and the highest possible value corresponding to 100 (Danielsson et al., 2017; Fayers et al., 2001).

Data were analysed using Statistical Program R. Demographic characteristics and the scores of the patient safety culture dimensions were summarized using descriptive statistics. Overall differences in the 12 dimensions between the four countries were tested using the Kruskal–Wallis test. If *p* < .05 was achieved the test was considered significant and a pairwise Wilcoxon test was performed to analyse any group difference. A multiple logistic regression analysis was performed to determine the association between the 12 dimensions and the four countries involved (explanatory variables), with countries x dimension as an interaction term and the overall patient safety grade (outcome variable). When there was an overall difference (*p *< .05), a pairwise test was performed with a Bonferroni correction (*p *< .01 was considered significant).

## 3 RESULTS

The sample in this study consisted of 393 European cancer nurses. Most of these cancer nurses worked in Germany (*N* = 160 [41%]), followed by the United Kingdom (*N* = 94 [24%]), the Netherlands (*N* = 74 [19%]) and Estonia (*N* = 64 [16%]). Most respondents worked in a Department of Medical Oncology (*N* = 171 [77%]), or Cancer Surgery (*N* = 22 [10%]). One third of respondents had professional experience of ≤5 years, one‐third between 6–15 years and a final third had worked as a nurse for more than 15 years. Furthermore, most participants had worked <5 years in the current area (56%), had direct patient contact and worked less than 40 hr a week (60%, Table 1). Cronbach’s alpha of each dimension was acceptable, except the dimension of “staffing” scored lower (0.53–0.66) in all four countries.

**Table 1:** Respondent characteristics

	All participants (*N* = 393)	Estonia (*N* = 64)	Germany (*N* = 160)	Netherlands (*N* = 74)	United Kingdom (*N* = 95)	*p*‐value
Primary working area, *n* (%)						
Medical oncology	171 (77)	40 (63)	–	60 (87)	71 (81)	.0018
Surgery	22 (10)	11 (17)		7 (10)	4 (5)	
Others	28 (13)	13 (20)		2 (3)	13 (15)	
Professional experience, *n* (%), years						
≤5	83 (37)	22 (34)	–	25 (35)	36 (40)	.089
6–15	76 (34)	23 (36)		31 (44)	22 (24)	
>15	67 (30)	19 (27)		15 (21)	33 (36)	
Years in work area, *n* (%), years						
≤5	126 (56)	36 (56)	–	33 (46)	57 (63)	.111
6–15	65 (29)	21 (33)		26 (37)	18 (20)	
≥16	35 (15)	7 (11)		12 (17)	16 (18)	
Years in this hospital, *n* (%), years						
≤5	82 (36)	24 (38)	–	22 (31)	36 (40)	.019
6–15	71 (31)	27 (42)		17 (24)	27 (30)	
≥16	74 (33)	13 (20)		33 (46)	28 (31)	
Weekly work time, *n* (%), hours						
≤39	136 (60)	10 (16)	–	66 (93)	60 (66)	.002
>39	90 (40)	54 (84)		5 (7)	31 (34)	
Direct contact with patients, *n*(%)						
Yes	208 (93)	59 (92)	–	66 (97)	83 (91)	<.0001
No	15 (7)	5 (8)		2 (3)	8 (9)	

Note

*p*‐value of the background characteristics were calculated with chi square test.

### 3.1 Patient safety culture dimensions

The mean score for “overall patient safety grade” was 61.3 for the total sample, but this score varied statistically significantly between the countries, as tested with the Kruskal–Wallis test (*p *< .0001). Cancer nurses from Germany had the lowest score (mean 55.5), while cancer nurses from the United Kingdom scored the highest patient safety grade (mean 72.0, Table 2).

**Table 2:** Distribution of the dimensions of the HSPSC

	All participants (*N* = 393)	Estonia (*N* = 64)	Germany (*N* = 160)	Netherlands (*N* = 74)	United Kingdom (*N* = 95)	*p*‐value
Patient safety culture dimensions, mean (*SD*)						
Teamwork within units	69.4 (17.0)	69.2 (19.1)	65.1 (16.2)	72.0 (15.3)	74.8 (16.0)	<.0001
Supervisor/manager expectations and actions promoting safety	63.0 (20.1)	67.8 (17.2)	59.2 (22.2)	56.9 (15.8)	72.0 (17.2)	<.0001
Organizational learning	64.8 (16.0)	66.7 (13.9)	62.3 (17.2)	62.6 (13.3)	69.5 (16.2)	.0004
Management support for patient safety	54.5 (19.8)	59.0 (17.6)	48.5 (22.1)	54.8 (6.1)	61.7 (20.7)	<.0001
Overall perception of patient safety	58.2 (17.6)	61.6 (15.5)	54.6 (17.4)	60.3 (17.5)	60.7 (18.4)	.003
Feedback and communication about errors	64.5 (19.8)	63.0 (20.2)	62.1 (20.8)	67.0 (14.9)	67.6 (18.4)	.156
Communication openness	63.1 (17.6)	65.6 (17.5)	60.5 (18.6)	60.1 (14.9)	67.5 (17.2)	.006
Frequency of events reported	56.7 (23.6)	44.6 (23.1)	51.1 (22.9)	63.0 (18.9)	69.5 (21.0)	<.0001
Teamwork across units	54.6 (16.1)	58.0 (14.8)	55.1 (14.7)	52.1 (18.9)	53.2 (16.6)	.223
Staffing	46.6 (18.6)	48.0 (19.1)	43.8 (18.9)	47.8 (15.1)	49.6 (19.1)	.178
Handoffs and transitions	47.9 (16.1)	46.4 (14.1)	48.1 (15.4)	45.2 (13.4)	51.0 (19.7)	.045
Non‐punitive response to errors	57.7 (19.7)	56.6 (18.9)	60.1 (20.0)	53.2 (14.6)	57.7 (22.7)	.021
Overall patient safety grade, mean (*SD*)	61.3 (18.7)	61.3 (16.6)	55.5 (18.6)	60.4 (15.6)	72.0 (17.8)	<.0001

Note

*p*‐value patient safety culture was calculated with Kruskal–Wallis test.

Overall, the highest rated patient safety culture dimensions were “teamwork within units” (mean 69.4), “organizational learning” (mean 64.8) and “feedback and communication about errors” (mean 64.5). The lowest rated dimensions were “handoffs and transitions” (mean 47.9) and “staffing” (mean 46.6, Table 2). Various dimensions of patient safety culture were rated differently in statistically significantly ways between the four countries. Cancer nurses from the United Kingdom scored higher than cancer nurses from the three other countries in most dimensions. This was especially evident on the dimensions “teamwork within units”, “supervisor/manager expectations and actions promoting patient safety”, “management support for patient safety” and “frequency of events reported” (Table 1). In all four countries, the dimension of staffing scored very low. This was mainly explained by the items that indicated that in all countries there were not enough cancer nurses available to handle the workload and that they were working in a “crisis mode” most of the time (File S1).

**Figure 1:**
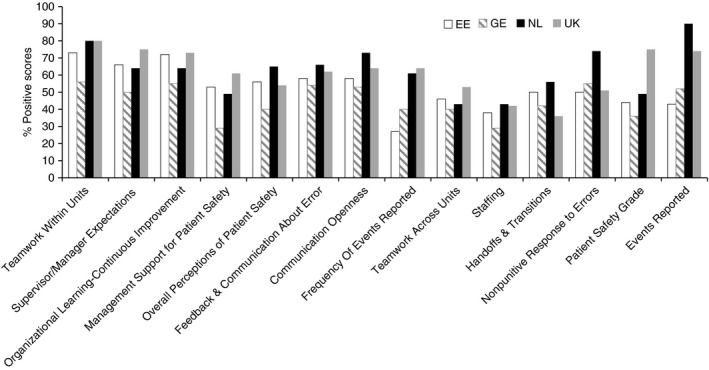
Percentage of the positive scores of the patient safety culture dimensions and single‐items per country

On the other hand, when looking at the percentages of positive answers overall, cancer nurses from the Netherlands scored the highest on “events reported”, “communication openness” and “non‐punitive response to errors”. Whereas cancer nurses from the United Kingdom offered the most positive perceptions of the single item question “overall patient safety grade”. Cancer nurses from both the United Kingdom and the Netherlands scored higher on the dimension “frequency of events reported” than cancer nurses from Estonia or Germany (Figure 1).

### 3.2 Factors associated with overall patient safety grade

Multiple logistic regression analysis showed a statistically significant difference between the four countries for associations between five of 12 dimensions of patient safety culture with the overall patient safety grade: overall perception of patient safety, communication openness, staffing, handoffs and transitions and non‐punitive response to errors (Table 3). A higher level of these five dimensions of patient safety culture implies increased probability for a higher overall patient safety grade. We found a statistically significant difference for the “overall perceptions of patient safety” between the four countries. This was mainly explained by the difference between Estonia and Germany (odds ratio [OR] 0.92, 95% confidence interval [CI] 0.865–0.969, pairwise Wilcoxon test: *p *< .002), meaning that for every increase in the “overall perceptions of patient safety” the change of having a higher “patient safety grade” was lower for Estonia compared with Germany. The statistically significant difference for “communication openness” was also explained by the differences between Estonia and Germany (OR [95% CI] = 0.93 [0.89–0.97], pairwise Wilcoxon test: *p *< .001). The statistically significant difference for “staffing” was explained by two comparisons, both between Estonia and Germany (OR [95% CI] = 0.92 [0.88–0.96], pairwise Wilcoxon test: *p *< .001) and the United Kingdom and Germany (OR [95% CI] = 0.95 [0.91–0.99], pairwise Wilcoxon test: *p *< .001). While the difference for “handoffs and transitions” was mainly explained by the difference between the Netherlands and Germany (OR [95% CI] = 0.93 [0.89–0.98], pairwise Wilcoxon test: *p *< .006). In the multiple logistic regression analysis, we found a *p *= .024 for “non‐punitive response to errors”. However, all pairwise comparisons showed a *p *> .01, due to the Bonferroni correction, meaning there is no significant difference between any of the countries for this variable.

**Table 3:** The association between the dimensions of the HSPSC and countries (explanatory variables) and overall patient safety grade

Dimensions	*p* a
Teamwork within units	.087
Supervisor/manager expectations and actions promoting safety	.097
Organizational learning	.301
Management support for patient safety	.150
Overall perception of patient safety	.003
Feedback and communication about errors	.073
Communication openness	.007
Frequency of events reported	.138
Teamwork across units	.074
Staffing	<.0001
Handoffs and transitions	.022
Non‐punitive response to errors	.024

a Multiple logistic regression analysis.

## 4 DISCUSSION

To the best of our knowledge, this is the first study comparing patient safety culture perceptions between cancer nurses in Estonia, Germany, the Netherlands and the United Kingdom. We demonstrated a statistically significant difference in the “overall patient safety grade” between the four countries. This was mainly explained by a statistically significant difference in the following dimensions of safety culture as measured by the HSPSC; overall perception of patient safety, communication openness, staffing, handoffs and transitions and non‐punitive response to errors.

One of the most important factors associated with a positive patient safety culture is openness in communication. This is not just about communication itself, but also related to nurses feeling confident to speak‐up and report unsafe events, without fear of negative consequences. Consequently, as team members nurses have the opportunity to learn from both their own and other’s mistakes, fostering a more open climate of organizational learning. In the current study, the nurses in the Netherlands and in the UK, scored higher on “communication openness”, the “frequency of events reported” and “non‐punitive response to errors” (Figure 1 and File S1). This is contrary with existing literature, where respondents in other studies scored remarkably lower on these items (Alswat et al., 2017; Blegen et al., 2009; Famolaro et al., 2016; Mardon, Khanna, Sorra, Dyer, & Famolaro, 2010). Our results may reflect the differences between the four countries in our study, especially in terms of contextual factors such as; recognition, autonomy, career opportunities, and educational preparation. Cancer nursing in both the Netherlands and the United Kingdom is recognized professionally and is well‐established as a distinct specialty in nursing. Both countries also have career possibilities in clinical cancer nursing, e.g. advanced nursing roles. However, in the other two countries this is not currently the case.

Patient safety is one of the most important factors in quality of care and is inseparable from the safety culture (Charalambous & Kelly, 2018; Ulrich & Kear, 2014). In the current study, one of the statistically significant factors related to patient safety culture was staffing. The percentage of positive responses, however, are lower for all staffing items, in all four countries (File S1; Figure 1). Earlier studies from the Arabic countries reported similarly low numbers (Alswat et al., 2017; El‐Jardali, Jaafar, Dimassi, Jamal, & Hamdan, 2010; Hamdan & Saleem, 2013), in contrast with studies from the USA and Sweden where respondents scored considerably higher regarding staffing (Blegen et al., 2009; Danielsson et al., 2017; Famolaro et al., 2016; Mardon et al., 2010). In our study, nurses from the Netherlands and the United Kingdom may be questioning whether there are enough nurses to guarantee patient safety, while in the other countries, the question is not only one of numbers, but also of the availability of suitably qualified specialist cancer nurses to promote a safety culture.

Earlier studies, such as the RN4CAST project, showed that nurses’ workload and level of education were directly linked with patient outcomes and, ultimately, with patient mortality (Aiken et al., 2014, 2017). Cancer nursing is developing to meet the rising demands of increased cancer incidence, prevalence and newer and more complex treatment options. The need for expertise in specialized and advanced cancer nursing is therefore also increasing (Charalambous et al., 2018). Some European countries have already implemented and seen the benefits from advanced cancer nursing roles to start meeting these rising needs (Cowman et al., 2010), while other do not yet recognize advanced nursing roles or even offer postgraduation education. To help countries to establish specialized education in cancer nursing, EONS published a Cancer Nursing Education Framework (2018), which describes the competences involved in cancer nursing and how these can be addressed in education programs globally.

### 4.1 Limitations

Although we used a validated questionnaire to measure patient safety culture, it is not always possible to compare these data to other studies. Most other studies include a variety of respondents such as managers, physicians, technicians, and sometimes but not always, nurses (Danielsson et al., 2017; Sorra et al., 2018). More importantly, such data are often reported in different ways. In some studies, the means of the 12 HSPSC dimensions are described as the mean of the Likert‐type scale (Burlison et al., 2016; Hammer et al., 2011; Smits et al., 2012), or a transformation of the Likert‐type scale into a 0–100 scale (Table 1; (Danielsson et al., 2017)). The disadvantage of using a mean is that it tends to shift towards the middle. Therefore, it does not always correctly reflect the range of opinions of all the respondents. In other studies, the percentage of positive answers for the 12 dimensions were reported (Figure 1; Alswat et al., 2017; Danielsson et al., 2017; Sorra et al., 2018), which may give a clearer reflection of the given responses. Our study was also carried out with a self‐selecting sample and may reflect the views of those motivated to attend professional conferences. Furthermore, we could not report on response rates. Wider samples and other recruitment strategies should be considered in future safety culture research studies involving cancer nurses.

This study presents an analysis of cancer nurses” opinions about the patient safety cultures in four European countries. Therefore, the results can only be seen as an indication about patient safety culture in these settings. Nevertheless, our data are derived from the responses of cancer nurses with a range of professional expertise and considerable years of experience of working in their current workplaces. As much as 93% of our respondents worked in direct contact with patients, representing what we suggest is a “true” range of clinical cancer nurses. Based on this study, we suggest that organizations need to ensure that cancer nurses are recognized as key members of multi‐professional care teams. This will require the further development of the status and recognition of cancer nursing in all countries to enhance the patient safety culture in cancer care.

## 5 CONCLUSION

This study showed that cancer nurses scored patient safety differently between four European countries. This is mainly explained by the dimensions of overall perception of: patient safety, communication openness, staffing, handoffs and transitions and non‐punitive response to errors.

As nurses are the largest group of professionals working directly with cancer patients the growing rates of cancer incidence and prevalence will increase the need for specialized cancer nursing roles in all countries. Nurses work closely with patients across the entire cancer trajectory and are therefore the best suited professionals to assess and promote the patient safety culture, however, all health professionals also have a responsibility in this regard.

As well as better awareness of patient safety generally there are also other issues now to be considered in practice contexts. These include having adequate numbers of Registered Nurses to meet the safety needs of patients with cancer. More research is now needed to examine cancer patient acuity in relation to nurse staffing levels and other factors such as the experience level and qualifications of the nurses available. Evidence already exists from international studies of the link between nurse staffing and nurse qualifications with patient mortality in acute medical and surgical units (Aiken et al., 2014, 2017).

Research effort should now be extended to oncology settings with agreed nurse‐specific outcomes being identified that reflect the needs of cancer patients such as infection, nausea, and vomiting or pain (Aiken et al., 2017; Oldenmenger et al., 2018). By doing so the safety culture of cancer settings will be enhanced by improving the visibility of the contribution made by cancer nurses and ensuring that they feel confident about speaking up when safety concerns arise.

## CONFLICTS OF INTEREST

No conflict of interest has been declared by the authors.

## AUTHOR CONTRIBUTIONS

All authors made substantial contributions to conception and design, or acquisition of data, or analysis and interpretation of data; involved in drafting the manuscript or revising it critically for important intellectual content; given final approval of the version to be published. Each author should have participated sufficiently in the work to take public responsibility for appropriate portions of the content; agreed to be accountable for all aspects of the work in ensuring that questions related to the accuracy or integrity of any part of the work are appropriately investigated and resolved.
