# Supplementary material for: Patient safety culture among European cancer nurses—An exploratory, cross‐sectional survey comparing data from Estonia, Germany, Netherlands, and United Kingdom
Source: J Adv Nurs. 2019 Sep 4;75(12):3535–43. doi: 10.1111/jan.14177 (PMC6899826; doi:10.1111/jan.14177)
Supplement: Supplementary file 1 [file JAN-75-3535-s001.docx]

**Supplementary file 1 for online only: Overall analysis of the positive scores**

|  | **EE**  **N=64, %** | **GE**  **N=160, %** | **NL**  **N=74, %** | **UK**  **N=95, %** | **P-value** |
| --- | --- | --- | --- | --- | --- |
| **Teamwork within units**  People support one another in this facility  When a lot of work need to be done quickly, we work together as a team to get the work done  In facility, people treat each other with respect  When one area in this unit gets really busy, others help out | 83  77  64  67 | 66  63  51  44 | 93  80  90  56 | 92  87  82  60 | <.0001  .0002  <.0001  .0054 |
| **Supervisor/ manager expectations and actions promoting safety**  My supervisor says a good work when he sees a job done according to established PS procedures  My supervisor seriously considers staff suggestions for improving patient safety  Whenever pressure builds up, my supervisor wants us to work faster, even if it means taking  shortcuts  My supervisor overlooks patient safety problems that happen over and over | 68  70  54  70 | 48  58  38  57 | 53  75  63  65 | 74  80  70  76 | .0003  .0021  <.0001  .02 |
| **Organizational learning**  We are actively doing thing to improve patient safety  Mistakes have led to positive changes here  After we make changes to improve patient safety, we evaluate their effectiveness | 87  52  63 | 75  36  53 | 73  61  41 | 89  64  65 | .007  <.0001  .001 |
| **Management support for patient safety**  Hospital management provides a work climate that promotes patient safety  The actions of hospital management show that patient safety is a top priority  Hospital management seems interested in patient safety only after an adverse event happens | 67  50  41 | 28  27  31 | 68  46  46 | 79  63  42 | <.0001  <.0001  .021 |
| **Overall perception of patient safety**  It is just by chance that more serious mistakes don’t happen around here  Patient safety is never sacrificed to get more work done  We had patient safety problems in this unit  Our procedures and systems are good at preventing errors from happening | 54  60  52  56 | 46  27  48  40 | 54  -  78  50 | 45  50  59  64 | .443  <.0001  .0066  .002 |
| **Feedback and communication about errors**  We are given feedback about changes put into places based on event reports  We are informed about errors that happen in this unit  In this unit, we discuss ways to prevent errors from happening again | 44  68  60 | 44  53  64 | 63  59  76 | 56  63  67 | .0072  .1912  .247 |
| **Communication openness**  Staff will speak up if they see something that may negatively affect patient care  Staff feel free to question the decisions or actions of those with more authority  Staff are afraid to ask questions when something does not seem right | 84  29  60 | 56  45  59 | 86  67  68 | 69  52  71 | <.0001  .0002  .1926 |
| **Frequency of events reported**  When a mistake is made, but is caught and corrected before affecting the patient, how often is  this reported?  When a mistake is made, but has no potential to harm the patient, how often is this reported?  When a mistake is made that could harm the patient, but does not, how often is this reported? | 24  22  34 | 41  27  51 | 49  58  75 | 47  67  79 | .0154  <.0001  <.0001 |
| **Teamwork across units**  Hospital units do not coordinate well with each other  There is good cooperation among hospital units that need to work together  It is often unpleasant to work with staff from other hospital units  Hospital units work well together to provide the best care for patients | 33  61  34  56 | 26  40  54  41 | 25  49  -  54 | 31  51  69  63 | .0327  .6064  .0002  .0076 |
| **Staffing**  We have enough staff to handle the workload  Staff in this unit work longer hours than is best for patient care  We use more agency/ temporary staff than is best for patient care  We work in ‘crisis mode’ trying to do too much, too quickly | 38  33  55  29 | 12  29  59  15 | 33  28  66  39 | 36  28  66  38 | <.0001  .9247  .3877  <.0001 |
| **Handoffs and transitions**  Things ‘fall between the cracks’ when transferring patients from one unit to another  Important patient care information is often lost during shift changes  Problems often occur in the exchange of information across hospital units  Shift changes are problematic for patients in this hospital | 50  67  20  62 | 22  48  34  64 | 32  58  32  54 | 29  51  27  38 | .0007  .1239  .2136  .0014 |
| **Nonpunitive response to errors**  Staff feel like their mistakes are held against them  When an event is reported, it feels like the person is being written up, not the problem  Staff worry that mistakes they make are kept in their personnel file | 31  61  59 | 48  54  64 | 70  75  78 | 59  55  40 | <.0001  .0175  <.0001 |
| **Patient safety grade** | 44 | 36 | 49 | 75 | <.0001 |
| **Number of Events reported** | 43 | 52 | 90 | 74 | <.0001 |

P-values were calculated with the Kruskal-Wallis test
